# Emerging Insights into the Role of BDNF on Health and Disease in Periphery

**DOI:** 10.3390/biom14040444

**Published:** 2024-04-05

**Authors:** Mayuko Ichimura-Shimizu, Khuleshwari Kurrey, Misaki Miyata, Takuya Dezawa, Koichi Tsuneyama, Masami Kojima

**Affiliations:** 1 Department of Pathology and Laboratory Medicine, Tokushima University Graduate School, 3-18-15 Kuramoto-cho, Tokushima 770-8503, Japan; ichimura.mayuko@tokushima-u.ac.jp (M.I.-S.); tsuneyama.koichi@tokushima-u.ac.jp (K.T.); 2 Department of Neuroscience, School of Medicine, Yale University, New Haven, CT 06520, USA; khuleshwari.kurrey@yale.edu; 3 Department of Applied Bioscience, College of Bioscience and Chemistry, Kanazawa Institute of Technology, 3-1 Yatsukaho, Hakusan 924-0838, Japan; b1925115@planet.kanazawa-it.ac.jp (M.M.); c6302229@st.kanazawa-it.ac.jp (T.D.)

**Keywords:** brain-derived neurotrophic factor, steatohepatitis, MASH, MASLD, metabolic syndrome, hyperphagia, inflammation, fibrosis, animal model

## Abstract

Brain-derived neurotrophic factor (BDNF) is a growth factor that promotes the survival and growth of developing neurons. It also enhances circuit formation to synaptic transmission for mature neurons in the brain. However, reduced BDNF expression and single nucleotide polymorphisms (SNP) are reported to be associated with functional deficit and disease development in the brain, suggesting that BDNF is a crucial molecule for brain health. Interestingly, BDNF is also expressed in the hypothalamus in appetite and energy metabolism. Previous reports demonstrated that BDNF knockout mice exhibited overeating and obesity phenotypes remarkably. Therefore, we could raise a hypothesis that the loss of function of BDNF may be associated with metabolic syndrome and peripheral diseases. In this review, we describe our recent finding that BDNF knockout mice develop metabolic dysfunction-associated steatohepatitis and recent reports demonstrating the role of one of the BDNF receptors, TrkB-T1, in some peripheral organ functions and diseases, and would provide an insight into the role of BDNF beyond the brain.

## 1. Neurotrophins

The secretion of diffusible molecules is a cell-extrinsic mechanism that plays a critical role in the structural and functional development of vertebrates. Neurotrophins are a family of structurally related diffusible molecules that exert many functions, including neuronal survival and differentiation, synapse formation and synaptic plasticity. One of the first neurotrophins to ever be described is the nerve growth factor (NGF). It was found to be expressed in the mouse submandibular gland and has been extensively characterized as a neuronal growth and survival-promoting protein [1,2]. However, in the central nervous system (CNS), only a few neuronal populations are responsive to NGF [3].

The second neurotrophin to be discovered was brain-derived neurotrophic factor (BDNF) and it was described by Barde and colleagues as a growth factor that can enhance the survival and differentiation of developing neurons [4,5,6,7]. In the adult brain, BDNF has a modulating role in synaptic function [8]. BDNF elicits synaptic transmission and plasticity in in vitro and in vivo experiments [8]. BDNF expression increased after learning tasks such as contextual fear conditioning, and genetic approaches using BDNF knock-out (KO) mice indicate that BDNF impairment affects learning tasks such as fear conditioning-dependent memory [9]. Moreover, three primary mechanisms exist for BDNF to control synapse development: enhancing axon and dendritic arborization, stimulating the production of axonal and dendritic boutons, and maintaining pre-existing synapses [10,11]. Additionally, BDNF has a temporally and spatially relevant effect on synaptogenesis [12]. Further, PCR technology identified two additional neurotrophins called neurotrophin-3 (NT-3) [13] and neurotrophin-4/5 (NT-4/5) [14]. They have been reported to have various biological roles like NGF and BDNF.

Interestingly, single nucleotide polymorphisms (SNP) are in the gene sequence encoding the BDNF precursor protein, and we reported that an SNP that converts Val to Met lowered the neuronal activity-dependent secretion of BDNF from neurons and that the SNP affects episodic memory in humans [15]. Since this SNP (Val66Met) is in the pro-domain of the BDNF precursor, we further investigated the biological role of the domain. We demonstrated that the pro-domain (BDNF pro-peptide) is a releasable protein and promotes long-term depression (LTD) of synaptic plasticity [16]. Furthermore, the BDNF pro-peptide was detected in cerebrospinal fluid, and the content was reduced in samples from patients with depression and schizophrenia [17], indicating that the BDNF research, including our studies, might contribute to the treatment and diagnosis of brain disorders. 

In addition to these mammalian neurotrophin members, NT-6 and NT-7 were identified from *Xiphophorus* and *Cyprinus carpio*, respectively [18,19]. NT-6 is more homologous to NGF than to other neurotrophins. NT-7 shares about 66% of its amino acid identity with Xiphophorus NGF and NT-6. It was shown that NT-7 is expressed in peripheral tissues much more than in the brain, suggesting that NT-6 and NT-7 might exert neurotrophin activity in peripheral tissues of lower vertebrates. Although it is less potent, recombinant pure NT-6 acts on chick sympathetic and sensory neurons in a similar fashion as NGF. The embryonic valvulla cerebelli expresses NT-6, which continues to be expressed in certain adult tissues. Its actions in the nervous system may be regulated by the way NT-6 interacts with molecules that bind heparin.

## 2. Neurotrophin Receptors for Their Biological Actions

Neurotrophins, consistent with other growth factors, initiate their biological actions through receptors. A previous report shows that NGF was internalized by receptor-dependent processes and transported along axons in membranous vesicles to the cell soma [20]. The identification of neurotrophin receptors and their downstream transmitter molecules that mediate the action of neurotrophins was revealed by numerous studies [21,22,23]. 

**Figure 1:**
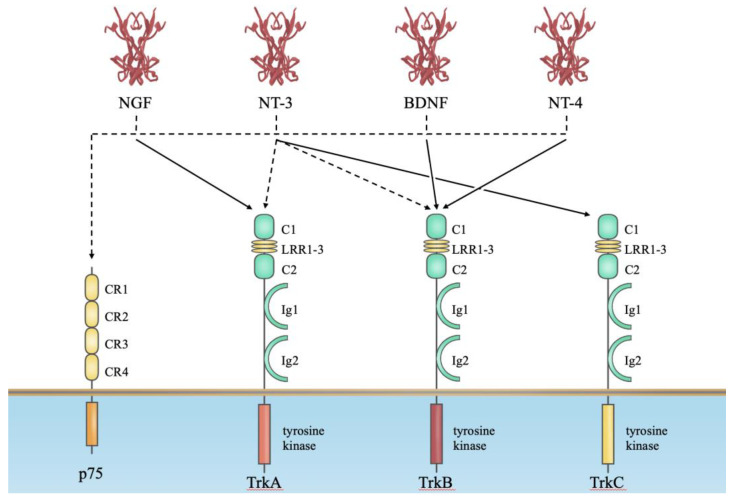
Neurotrophin–receptor interactions. This diagram shows the relationship between four mammalian neurotrophins and their receptors. All neurotrophins bind with low affinity to p75, as indicated by the dashed lines in the figure. However, NGF, BDNF, NT-3 and NT-4/5 can bind more specifically and with higher affinity to TrkA, TrkB and TrkC, as shown by the solid line. Here, we depict only the major tyrosine kinase-containing isoforms of the Trk receptor. However, more details on what molecules bind to the intracellular domain and exert neurotrophin action after binding can be documented in the references cited in the text [21,22,28].

Neurotrophins bind to two classes of transmembrane receptor proteins, the Trks (tropomyosin receptor kinases) and the neurotrophin receptor p75 [21] (Figure 1). This dual system allows for contrasting signaling after ligand binding, such as cell death signaling by p75 and cell survival signaling by Trk receptors [21]. These two classes of receptors have also been reported to interact directly. The Trk receptors belong to the family of receptor tyrosine kinases, and three independent *Trk* genes have been identified. NGF is the preferred ligand for TrkA, BDNF and NT4/5 are preferred for TrkB, and NT3 is preferred for TrkC [23]. Their tyrosine kinase domains are highly homologous (shared identity of ∼80% amino acids), whereas the extracellular domains are divergent in the amino acid sequence (∼30% shared). p75 belongs to a large family of TNF receptors [21]. Notably, p75 by itself does not exert the kinase-like activity seen in the Trk receptor. However, p75 has been revealed to transduce intracellular signals by associating or dissociating with cytoplasmic molecules, including enzymes and interacting factors [21]. The binding affinity of neurotrophins for their receptors was previously determined [21]. As shown in Figure 1, neurotrophins (NGF, BDNF, NT-3 and NT-4/5) bind to Trk with high affinity and p75 receptors with low affinity (approximately 10^−11^ M [Trk receptor] and 10^−9^ M [p75 receptor]). Therefore, such differences in binding affinity and downstream molecules between Trk receptors and p75 might produce different biological actions.

Since this review article focuses on the role of the BDNF-TrkB complex in peripheral diseases, we describe the TrkB receptor isoforms produced by alternative splicing [24]. Although the TrkB locus can produce many different TrkB receptor isoforms by alternative splicing, the most abundant isoform type is TrkB-FL, which includes the intracellular catalytic tyrosine kinase domain and TrkB-T1 lacking the kinase domain. Developmentally, TrkB-FL is predominantly expressed in the embryonic to early postnatal CNS. However, TrkB-T1 expression is very low in the embryo but increases postnatally and peaks in adulthood [25]. Lastly, some reports show that TrkB.T1 is the main isoform in peripheral tissues [26,27]. TrkB-T1 comprises exons up to exon 15 that encode the extracellular, transmembrane, and juxtamembrane domains shared by the TrkB-FL receptor. The splicing machinery employs exon 16, which encodes a short intracellular 11 amino acid tail (FVLFHKIPLDG) that is specific to the TrkB-T1 isoform after exon 15, rather than exon 17, the first exon specific to the TrkB-FL isoform. Although this sequence is 100% conserved across rats, humans, and chickens, it shows no apparent resemblance to known protein motifs. Exon 16 has a stop codon, its 3′UTR region with multiple polyadenylation sites, and an 11 aa tail. It is unknown what mechanism drives the alternative splicing that codes for the TrkB-T1 isoform compared to the TrkB-FL isoform.

## 3. BDNF Is a Regulator of Energy Balance, Body Weight and Food Intake

An interplay of hormones, brain circuits and peripheral tissues regulates the relationship between energy intake and expenditure. Several factors have been demonstrated to have an essential role in the central control of energy balance, including leptin, melanocortin and dopamine [29,30,31]. The initial report indicating that BDNF plays a vital role in controlling body weight and energy metabolism in the periphery came from the study of a chronic intracerebroventricular infusion of recombinant human BDNF [32]. Although this report examined the effect of BDNF on the function of cholinergic neurons, it was found that the body weight of adult rats was decreased [32]. This pharmacological finding aligned with genetic evidence collected from genetically engineered mutant mice. *Bdnf*-heterozygous mice exhibited increased body weight and mild hyperphagia [33,34]. The link between TrkB signaling and energy imbalance is also elucidated by studies using Cre transgenes to generate genetically engineered mice. It was reported that mutations in the genes encoding BDNF and its receptor TrkB lead to severe obesity in humans [35,36,37,38]. 

The relationship between food restriction and BDNF gene expression has also been studied, and it was shown that food deprivation for 2 days elicited a dramatic and selective reduction in *Bdnf* mRNA levels in the ventral medial hypothalamus (VMH) of mice and *Bdnf* mRNA levels in the VMH increased when melanocortin or glucose was administered to fasted mice [39,40,41], suggesting that both are actively involved in the energetic state and *Bdnf* gene expression in the VMH. This relationship between feeding and energy metabolism and BDNF is detailed in a review article written by Xu and Xie [42]. 

## 4. Role of BDNF in Insulin-Secretion Mechanism and Exercise

Mice and humans with mutations in BDNF or TrkB develop hyperappetitive obesity [43,44] due to impaired hypothalamic anorexia and hypercompetitive signaling pathways [43,45]. However, the pathogenesis of these peripheral symptoms is largely unknown, and the role of BDNF/TrkB in the periphery has been under investigation. An interesting finding is that systemic injection of BDNF improves blood glucose levels and alleviates fasting hyperglycemia in an obese mouse model [46]. 

Lino Tessarollo’s group reported a novel mechanism for regulating glucose homeostasis via peripheral action of BDNF. They first showed that the BDNF receptor TrkB-T1 was expressed in pancreatic beta cells and required for the secretion of insulin. The role of this body hormone is to decrease blood glucose levels, and it is secreted from pancreatic β-cells through depolarization caused by glucose uptake and metabolism. Notably, the signaling of BDNF-TrkB-T1 triggered calcium release from intracellular stores and increased glucose-induced insulin secretion. This was further demonstrated by the manifestation of an impairment in glucose tolerance and insulin secretion in mice lacking TrkB-T1 [47]. Interestingly, it was reported that exercise-induced BDNF expression in skeletal muscle [48,49]. This organ may have the potential to be a primary source of systemic BDNF because of the large body skeletal muscle mass. However, there is not yet confidential evidence that skeletal muscle can secrete BDNF.

## 5. Metabolic Syndrome and Steatohepatitis

In the mature brain, BDNF plays a vital role in forming neural circuits which impacts learning and memory. As we age, humans become more susceptible to various diseases including lifestyle-related diseases and metabolic syndrome. However, the role of BDNF over the long-life course still needs to be fully understood. We recently reported that reduced BDNF is associated with the development of one of the metabolic diseases, metabolic dysfunction-associated steatohepatitis (MASH), in two independent genetically engineered mouse lines with reduced BDNF expression [50]. 

Metabolic dysfunction-associated steatotic liver disease (MASLD), formerly known as non-alcoholic fatty liver disease (NAFLD), is one of the most prevalent diseases worldwide, affecting more than 30% of the global population [51,52]. MASLD encompasses a range of disease states, from isolated lipid accumulation or steatosis to its active inflammatory form MASH; formerly named nonalcoholic steatohepatitis (NASH) [53]. MASH is defined histologically as the presence of lobular inflammation, ballooning (or cellular) degeneration and fibrosis in addition to steatosis in the liver, and may advance to end-stage liver disease, including liver cirrhosis and hepatocellular carcinoma [54,55]. The pathological characteristics of steatohepatitis comprise the following: infiltration of inflammatory cells, particularly neutrophils, into the hepatic parenchyma; ballooning degeneration of injured hepatocytes; and hepatocellular steatosis extending from the centrilobular/perivenular region to the distal region. Fibrosis begins in the centrilobular/perivenular area, passing through portal and sinusoidal fibrosis, bridging fibrosis, and nodule development.

MASLD/MASH is a complex and heterogeneous disease involving many genetic, epigenetic, and environmental factors. MASLD is commonly associated with obesity, type 2 diabetes mellitus, hypertension, hyperglycemia, and hyperlipidemia. Thus, MASLD/MASH is considered to be a hepatic phenotype of metabolic syndrome. In order to understand the pathogenesis of the highly diverse disease states of MASLD/MASH, the multiple parallel hit hypothesis serves as a valuable model suggesting that an interplay between the liver and other extrahepatic organs such as the adipose tissue and the intestine, in addition to interactions that exist within the liver itself, all contribute significantly to the pathogenesis of MASLD/MASH [56,57]. The adipose tissue, along with the liver, regulates energy homeostasis through the modulation of fatty acid metabolism [57]. When adipocytes are enlarged due to obesity, the adipose tissue secretes inflammatory cytokines and chemokines such as tumor necrosis factor (TNF)-α, interleukin (IL)-6, and leptin, along with infiltration of immune cells, including macrophages [58,59]. The inflammatory state in adipose tissue induces systemic inflammation, which may exacerbate liver disease, exemplifying an adipose tissue–liver crosstalk. Diets rich in fat, fructose, and cholesterol, known as Western diets, have a direct impact on liver metabolism, while dietary components induce structural and functional changes in gut microbiota composition, which is referred to as dysbiosis, and contribute to liver disease [56,60,61]. Pro-inflammatory dietary factors and dysbiosis induce intestinal inflammation, resulting in an impaired intestinal permeability that promotes the translocation of bacteria components, such as lipopolysaccharide, and bacteria-derived metabolites to the liver via the portal vein [60]. 

Even though there is increasing evidence that elucidates the mechanistic pathogenesis of MASLD/MASH, there are no Food and Drug Administration-approved pharmacological interventions for MASLD/MASH [62]. It is possible that the diversity of pathogenic factors in MASH may not allow focusing on the potential therapeutic target, leading to some patients not showing any effect of the drug even if they have the same histological phenotype in the liver. Expanding observation of the events beyond the intestine and adipose tissue with appropriate mouse models will facilitate understanding of the intra/extrahepatic pathogenesis of the disease.

## 6. Animal Models of MASLD/MASH

Using animal models is attractive for elucidating pathogenic pathways in which multiple organs interact complexly. Compared to clinical research, animal research does not have as many ethical restrictions when it comes to obtaining liver and other organ samples. The ideal animal model of MASLD/MASH should accurately reflect both the pathology of steatohepatitis and the context within which it develops. Specifically, the liver phenotype in animal models of MASLD/MASH should present with steatosis, lobular inflammation, ballooning degeneration and fibrosis. The histopathological pattern should be a central lobular lesion as seen in MASH in humans. Also, the development of obesity and insulin resistance, which are the underlying pathologies of MASLD/MASH, is highly desirable. Furthermore, it is preferable for the model to exhibit an array of comorbidities such as dyslipidemia and adipose tissue inflammation, both of which can act as a cause and a consequence of the development and progression of MASLD/MASH [63]. To meet these requirements, animal models have been established through genetic manipulation, modified diets, and combinations of the two [64]. Representative MASLD/MASH rodent models are listed in Table 1, with a focus on those caused by overnutrition and unbalanced diets.

Since the development of MASLD/MASH is linked to high-calorie diets and excessive consumption of cholesterol or fructose, diet-focused animal models mainly utilize methionine choline-deficient diet (MCD) and high-fat diet (HFD). A standard MCD is high in sucrose (40% by weight) and fat (10–40% fat kcal), and is deficient in choline and methionine, which are essential for fatty acid β oxidation and production of very low-density lipoprotein [65,66,67]. The MCD model has been used most classically and presents severe liver pathology in a short period of time: steatosis and steatohepatitis in 2–4 weeks and even fibrosis in 8–10 weeks with high reproducibility despite the fact that rodents are characteristically resistant to fibrosis [63,64]. However, weight loss and a lack of insulin resistance make the MCD model quite different from human MASLD/MASH. In contrast, rodents on an HFD (32–60% fat kcal) mimic human MASH in liver histological phenotype as well as in weight gain and insulin resistance [68]. However, the pathological outcome is not severe, and the model is limited as it takes more than 6 months for mild fibrosis to develop [64,69]. Adding cholesterol and cholate to the HFD ameliorates that shortcoming and leads to the time-dependent development of progressive steatohepatitis and fibrosis over 6–24 weeks [63,70,71,72,73]. We have also been able to make a MASH mouse model that develops advanced fibrosis (stage 3–4) in a relatively short period of time, by formulating an iHFC diet, which is an HFD with well-calculated supplementation of cholesterol and cholate [74]. Previous studies have shown that the cholate content of HFD and the mouse strain can make differences in the rate and severity of fibrosis onset and in the occurrence of comorbid symptoms like obesity [75,76,77,78]. 

Genetic animal models of MASLD/MASH are particularly valuable for studying specific pathways of liver metabolism. Although alteration of specific genes can cause deviation in the pathogenic mechanisms in these models and in humans, the recapitulation of the systemic and hepatic phenotype induced by gene modification is appealing. Hyperphagic models can be MASLD/MASH models as well as models for obesity. Mice lacking either leptin (*ob*/*ob* mice) or the leptin receptor (*db*/*db*) are hyperphagic and develop severe obesity, insulin resistance, hyperlipidemia and hepatic steatosis, but rarely develop steatohepatitis without additional stimuli (HFD, MCD, etc.) [63,64]. However, *ob*/*ob* mice are resistant to fibrosis because leptin is an essential mediator of liver fibrogenesis [79,80]. Mice deficient in melanocortin 4 receptor (MC4R), which is expressed in the hypothalamic nuclei and regulates appetite, also develop hyperphagic obesity, insulin resistance, hyperlipidemia, and hepatic steatosis [81,82]. In addition, MC4R-deficient mice fed with an HFD for 20 weeks develop steatohepatitis and fibrosis that mimic the histological features of human MASH. These mice also spontaneously develop hepatocarcinoma one year post-treatment.

A wide variety of animal models have been reported in addition to the ones mentioned above, and many review articles point out that only a few models recapitulate all the key features of human disease [63,64]. This may be one reason for the delay in drug discovery for MASLD/MASH. Accurate evaluation and understanding of the pathology and underlying mechanisms of animal models is necessary in order to translate findings from the bench to the bedside.

## 7. BDNF Hypothesis in MASH

**Figure 2:**
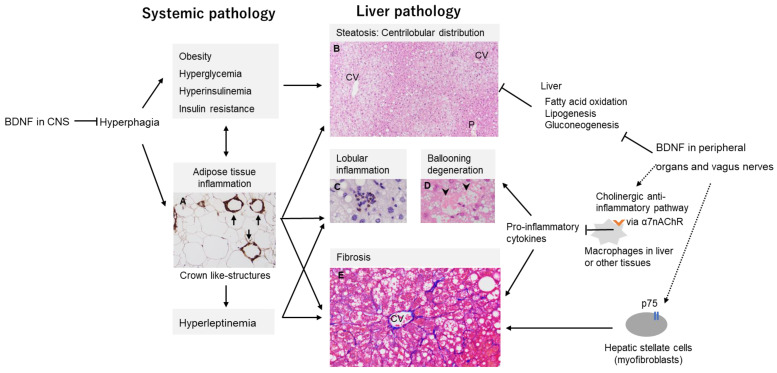
MASH pathology and possible pathogenic mechanism involving BDNF. Reduced BDNF expression induces steatohepatitis with typical systemic pathology in genetically engineered mice [50]. Steatohepatitis is characterized histologically by steatosis, lobular inflammation, ballooning degeneration of hepatocytes and fibrosis with obesity-associated metabolic dysfunction. The observed phenotype in these mice is indicated by gray-colored squares. Although the mechanism of action and source of BDNF affecting the liver are still uncertain, investigating two possible BDNF pathways, the central nervous system (CNS)-mediated pathway and the peripheral organs- and vagus nerve-mediated pathway, may help to better understand the pathology of MASH. (**A**). Adipose inflammation (represented by crown like-structures): Enlarged and dead adipocytes surrounded by macrophages (arrows) stained with the anti-Mac-2 antibody are signs of inflammation, which is one of the pathological phenotypes of obesity. (**B**). Hepatic steatosis: Micro-macrovesicular lipid droplets distribute predominantly to centrilobular/perivenular regions as seen via hematoxylin and eosin (H&E) staining. CV, central vein; P, portal tract. (**C**). Lobular inflammation: Inflammatory cells including lymphocytes and neutrophils infiltrate the hepatic parenchyma. Neutrophils were immunohistochemically stained with an anti-myeloperoxidase antibody. The presence of neutrophils is one of the features of MASH histology. (**D**). Ballooning degeneration of hepatocytes: Hepatocytes degenerate like a balloon (arrowheads, H&E staining), which is one of the critical findings that distinguish MASH from other liver diseases. (**E**). Fibrosis: Delicate fibers which are colored in blue and similar in appearance to chicken wire, typically arise perivenularly/perisinusoidally in the centrilobular/perivenular region as seen via Azan-Mallory staining. α7nAChR, α7 nicotinic acetylcholine receptor.

In our recent study using genetically engineered mice that exhibit reduced levels of BDNF protein expression such as BDNF heterozygous (BDNF^+/−^) and proBDNF knock-in (BDNF^pro/+^) mice, in which the precursor BDNF (proBDNF) is inefficiently converted into the mature form of BDNF, we are the first to demonstrate that the mutant animals spontaneously develop the full spectrum of MASH, including both liver-specific lesions and systemic pathology such as obesity and insulin resistance (Figure 2) [50]. Based on the progressive liver lesions with fibrosis and the quite mimicked systemic metabolic dysfunction in these mice, we identified these two mice as novel models of MASH. In order to discuss MASH pathogenesis involving BDNF, it is necessary to consider both of the following possible mechanisms: the pathological phenotype of the liver might be one of the metabolic dysfunctions associated with overeating due to BDNF reduction in the brain (CNS-mediated effects of BDNF) or the phenotype might be induced by low BDNF expression in peripheral tissue other than the brain (non-CNS mediated effects of BDNF).

### 7.1. MASH Development by CNS-Mediated Effects of BDNF

Hyperphagia and subsequent obesity-associated metabolic disorders are risk factors for the development of MASH [64,83]. We previously found that BDNF heterozygous mice exhibited hepatic steatosis, one of the most frequent symptoms observed in obesity and that liver lesions of MASH coincide with increased levels of food intake, body weight, serum glucose and insulin. Serum leptin, an appetite suppressant hormone, was found to be elevated in these BDNF heterozygous mice. Nonetheless, excessive leptin secretion did not suppress appetite and induced obesity in BDNF heterozygous mice. The mechanism underlying this phenomenon can be the dependence of the normal action of leptin in the hypothalamus on *Bdnf* transcripts within neurons [84]. 

Meanwhile, the previous study showed that food restriction initiated at 6 weeks of age, a period at which obesity in BDNF heterozygous mice is not yet apparent, induced normalization of body weight, and serum glucose and insulin levels. As a result, hepatic steatosis was suppressed, suggesting that BDNF signaling in the CNS could contribute significantly to hepatic lipid metabolism. In contrast, lobular inflammation with neutrophil infiltration was still observed in the livers of food-restricted mice, appearing like typical early MASH histology. The presence of neutrophils in the liver is one of the hallmarks of MASH pathology [85,86]. Taken together, reduced BDNF expression could induce hepatic steatosis via CNS appetite-regulating pathways and could contribute occurrence of hepatic inflammation by non-CNS pathways (Figure 2). Given that there is a population of lean patients with MASH [87], it is speculated that there is a subtype of MASH that is independent of hyperphagia or obesity. In this context, BDNF might be a potential contributor to this subtype. 

### 7.2. Non-CNS Mediated Effects of BDNF on MASH Development

BDNF and its high-affinity receptor, TrkB, are widely expressed in peripheral organs including skeletal muscle, liver, pancreas, and adipose tissue [88]. BDNF in the skeletal muscle exerts its action such as lipid metabolism in an autocrine or a paracrine manner [89]. As skeletal muscle expresses BDNF second to the brain, the role of BDNF in both muscle and muscle–brain crosstalk has been elucidated [90,91]. In contrast, little is known about how BDNF affects liver tissue. It has been reported that intracerebroventricular BDNF administration suppresses hepatic gluconeogenesis and lowers blood glucose levels [92,93]. Moreover, this effect occurs independently of changes in food intake [92], suggesting BDNF can affect hepatic metabolism directly. This has been further corroborated by a study that demonstrated that BDNF enhanced fatty acid oxidation and inhibited lipogenesis and gluconeogenesis in hepatocytes (Figure 2) [94]. 

In the previous study, an interesting finding in the mice with low BDNF expression was the occurrence of hepatic inflammation as well as steatosis. The vagus nerves which carry signals between the brain, heart and digestive system have been reported to regulate innate immune responses and inflammation via cholinergic stimulation of the α7 nicotinic acetylcholine receptor (α7nAChR). This receptor is expressed on the surface of microglia and peripheral macrophages as well as in the postsynaptic neurons, and suppressively mediate the production of pro-inflammatory cytokines [95,96,97]. In a dietary model of MASH, hepatic α7nAChR deficiency has been reported to exacerbate inflammation and fibrosis through macrophage activation and excessive production of TNF-α and IL-6 in the liver [95,98]. Although it is currently unclear how BDNF is involved in the “cholinergic anti-inflammatory pathway” in MASH pathogenesis, it has been shown that BDNF regulates the expression levels of α7nAChR in the hippocampus [99,100]. Moreover, afferent vagal nerves express TrkB during development and in maturity, and BDNF knock-out results in a substantial loss of vagal afferents [101]. In the neonatal mouse stomach wall, BDNF deficiency has been reported to inhibit the survival or formation of vagal mechanoreceptors [101]. Importantly, the anti-inflammatory effect of the vagus nerve is bidirectional, i.e., the afferent vagals detect peripheral cytokines and transmit signals via the medulla to the hypothalamus, which then communicates via efferent vagals to suppress peripheral inflammation [96]. A decrease in BDNF, through attenuation of this inflammatory reflex, would probably lead to chronic inflammation such as what is observed in MASH (Figure 2).

The low-affinity nerve growth factor receptor of p75 is also expressed in the liver, especially in the stellate cells which are involved in fibrosis by generating collagen as myofibroblasts. It has been reported that NGF and its precursor NGF (proNGF) act on stellate cells via p75 to regulate differentiation into myofibroblasts and cell survival, although the BDNF effect has not been clarified [102]. Investigating the pathophysiological effects of BDNF with a focus on the various liver cells involved in MASH pathogenesis, such as stellate cells and macrophages, may provide more insight into the pathogenesis of non-hypothalamic-mediated liver injury. Furthermore, it is important to explore the role of BDNF on inflammatory responses in peripheral organs other than the liver, in accordance with a multiple parallel hits pathogenesis, since Teillon et al. reported that selective depletion of hepatic BDNF has no effect on hepatic steatosis or inflammation in mice [103].

### 7.3. Role of Chromatin Structure and Gene Transcription Mechanism of BDNF Gene in MASH Pathogenesis

Chromatin in eukaryotic cells is a higher-order structure that not only stores genomic information but also regulates the dynamics of accessibility to transcriptional control in response to external signals [44,45]. In addition, histone and transcription factors function in concert with DNA-modifying enzymes to provide regulation of fine-tuning of gene expression [46,48,49]. In the liver, gene expression is shown to be responsive to nutritional and hormonal signals, but transcriptomic and proteomic studies have demonstrated that genes whose expression is altered by diet-induced MASH include abnormalities in metabolic genes, inflammatory responses and induction of genes involved in fibrosis [104,105,106]. We recently reported that knockout mice of the neurotrophic factor BDNF develop MASH, and RNA-seq analysis revealed similar MASH pathogenesis-associated gene cluster changes as described in the above-mentioned paper, and a genetic polymorphism model of BDNF with an impaired precursor BDNF (proBDNF) processing response was found to develop MASH in mice [50]. Recent reports that polymorphisms at several loci correlate with human MAFLD risk [43,107] suggest a relationship between dysregulation of gene expression in chromatin and the development of MASH.

Xiong et al. recently reported that heterogeneous nuclear ribonucleoprotein U (hnRNPU), a nuclear matrix protein that regulates chromatin structure and gene transcription, links chromatin disruption and MASH development [108]. They showed that the nuclear matrix protein hnRNPU plays an essential role in the regulation of hepatic chromatin accessibility and gene expression in MASH, and that hepatocyte-specific inactivation of hnRNPU leads to an exacerbation of diet-induced MASH pathogenesis RNA-seq and chromatin immunoprecipitation. seq studies have shown extensive overlap between hnRNPU occupancy and changes in gene expression in MASH, suggesting that MASH may cause dynamic changes in chromatin structure [108]. They showed that hepatocyte-specific inactivation of hnRNPU disrupts liver chromatin accessibility, activates MASH molecular signatures, and sensitizes mice to diet-induced MASH pathology. hnRNPUs are also involved in the inactivation of MASH genes in the liver. And as a mechanism, we found that loss of hnRNPU stimulates the expression of TrkB-T1, which promotes inflammatory signaling and stress-induced cell death in hepatocytes and BDNF administration protected mice from diet-induced MASH by reducing membrane TRKB-T1 protein, promoting liver damage and the emergence of disease-specific signaling patterns that exacerbate the pathogenesis of MASH. These findings suggest that disruption of chromatin structure promotes liver damage and the emergence of disease-specific signaling patterns that aggravate the pathogenesis of MASH.

Of note, Xiong et al. showed that inactivation of the nuclear matrix protein hnRNPU, which is important for the regulation of chromatin structure and gene transcription, occurs in mouse hepatocytes with diet-induced MASH and can exacerbate the pathogenesis of MASH. Furthermore, the authors have identified a new nuclear molecular pathomechanism in MASH where inactivation of this hnRNPU protein leads to stress-induced cell death of hepatocytes via TrkB-T1. It is therefore of interest that their report is in line with our findings that MASH develops in BDNF-depleted mice, and these papers might implicate BDNF signaling with MASH pathology.

## 8. Conclusions

In addition to performing functions such as memory and learning, the brain also plays a vital role in receiving information from the body and governing the body. The importance of neurotrophic factors in such roles has been previously reported. Interestingly, BDNF, illustrated here, also contributes to feeding and energy metabolism. We recently demonstrated that BDNF-knockout mice develop MASH in the liver. This suggests that reduced BDNF expression may contribute to developing peripheral organ diseases with metabolic syndrome as a possible proximate risk factor. The elucidation of the mechanisms of MASH pathogenesis by reduced BDNF is a future research question to be investigated. One interesting hypothesis raised is that BDNF may play an important role in maintaining the brain–peripheral organ axis and in healthy aging and longevity.

## Figures and Tables

**Table 1:** Experimental animal models focused on those caused by overnutrition and unbalanced diets for the MASLD/MASH study.

Model	Metabolic Phenotype				Liver Histology		
	Obesity	Insulin Resistance	Adipose Tissue Inflammation	Dyslipidemia	Steatosis	Steato-Hepatitis	Fibrosis
Methionine and choline deficient diet	No (weight loss)	No	No (decreased adiposity)	No	Yes	Yes	Yes
HFD	Yes	Yes	Yes	Yes	Yes	Yes (mild)	Yes (mild)
HFD with cholesterol and cholate (iHFC diet)	Yes (depends on cholate-dose)	Yes	Yes	Yes	Yes	Yes	Yes
*ob*/*ob* mice	Yes	Yes	Yes	Yes	Yes	No (does not develop spontaneously)	No (resistant to fibrosis)
*db*/*db* mice	Yes	Yes	Yes	Yes	Yes	No (does not develop spontaneously)	No (does not develop spontaneously)
Melanocortin 4 receptor–deficient mice	Yes	Yes	Yes	Yes	Yes	Yes	Yes (under the HFD feeding)
BDNF knockout (+/−) mice	Yes	Yes	Yes	No	Yes	Yes	Yes
proBDNF knock-in mice	Yes	Yes	Yes	No	Yes	Yes	Yes

BDNF, brain-derived neurotrophic factor; HFD, high-fat diet; iHFC diet, one of the diets containing HFD supplemented with cholesterol and cholesterol.
